# Porcine Epidemic Diarrhea Virus and Discovery of a Recombinant Swine Enteric Coronavirus, Italy

**DOI:** 10.3201/eid2201.150544

**Published:** 2016-01

**Authors:** M. Beatrice Boniotti, Alice Papetti, Antonio Lavazza, Giovanni Alborali, Enrica Sozzi, Chiara Chiapponi, Silvia Faccini, Paolo Bonilauri, Paolo Cordioli, Douglas Marthaler

**Affiliations:** Istituto Zooprofilattico Sperimentale della Lombardia ed Emilia Romagna, Brescia, Italy (M.B. Boniotti, A. Papetti, A. Lavazza, G. Alborali, E. Sozzi, C. Chiapponi, S. Faccini, P. Bonilauri, P. Cordioli);; University of Minnesota Veterinary Diagnostic Laboratory, St. Paul, Minnesota, USA (D. Marthaler)

**Keywords:** Porcine epidemic diarrhea virus, coronavirus, viruses, Transmissible gastroenteritis virus, Porcine respiratory coronavirus, recombinant coronavirus, genetic recombinations, phylogeny, domestic pigs, swine, Italy

## Abstract

Porcine epidemic diarrhea virus (PEDV) has been detected sporadically in Italy since the 1990s. We report the phylogenetic relationship of swine enteric coronaviruses collected in Italy during 2007–2014 and identify a drastic shift in PEDV strain variability and a new swine enteric coronavirus generated by recombination of transmissible gastroenteritis virus and PEDV.

*Porcine epidemic diarrhea virus* (PEDV) and *Transmissible gastroenteritis virus* (TGEV) (family Coronaviridae, genus *Alphacoronavirus*) are enveloped viruses that contain a single-stranded, positive-sense RNA genome of ≈28 kb. Infection with these viruses causes watery diarrhea, dehydration, and a high mortality rate among suckling pigs. Coronaviruses (CoVs) are prone to genetic evolution through accumulation of point mutations and homologous recombination among members of the same genus (*1*). Porcine respiratory coronavirus (PRCV), a mutant of TGEV, appeared in pigs in the 1980s (*2*). The spread of PRCV, which conserved most of the antigenic sites and causes cross-protection against TGEV (*3*), led to the gradual disappearance of TGEV. Newly emerging CoVs pose a potential threat to human and animal health because multiple human CoV infections have been derived from animal hosts. Emerging swine coronaviruses are of great concern to swine health because of the potential increase in viral pathogenesis.

In 1978, PEDV was first identified in Europe; subsequent reports occurred in many countries in Asia, including China, Japan, Korea, and Thailand. In 2010–2012, genetically different PEDV strains emerged, causing severe outbreaks in China (*4*). PEDV spread to the United States, Canada, and Mexico in 2013–2014, and genetically related strains were detected in South Korea and Taiwan (*5**–**7**).* The PEDV outbreak caused large global economic losses to the swine industry. In Europe, a severe PEDV epidemic occurred in Italy during 2005–2006 (*8*), and in 2014–2015, PEDV was detected in Germany, France, and Belgium. These strains have a high nucleotide identity to PEDV strains that contain distinct insertions and deletions (INDELs) in the S gene (S-INDELs) from the United States (*9**–**11*). We report the detection and genetic characterization of swine enteric CoVs circulating in Italy during 2007–2014. We also report a recombinant TGEV and PEDV strain (identified as the species *Swine enteric coronavirus* [SeCoV]) circulating from June 2009 through 2012. Finally, we describe the phylogenetic relationship of the 2014 PEDV S-INDELs to the recent PEDV strains circulating in Europe.

## The Study

During 2007–2014, we collected 27 fecal and 24 intestinal samples from pigs with suspected PEDV or TGEV infections; the pigs came from swine farms in northern Italy (Po Valley), which contains the regions of Piemonte, Lombardia, Emilia Romagna, and Veneto (Technical Appendix Figure 1). The Po Valley contains 70% of Italy’s swine. Clinical signs included watery diarrhea in sows and a death rate in piglets of 5%–10%, lower than is typical with PEDV or TGEV infections. Samples were submitted for testing by electron microscopy, PEDV ELISA, viral isolation, pan-CoV reverse transcription PCR (RT-PCR), and RT-PCR for PRCV and TGEV; selected positive pan-CoV samples were sequenced (*12**–**14*) (Technical Appendix).

Results of electron microscopy showed that 25 (49%) of the 51 samples contained CoV-like particles, but all samples were negative for viral isolation. Although only 38 samples (74%) were positive by pan-CoV RT-PCR, 47 (92%) were positive by the PEDV ELISA (Table 1) (*12**,**13*). Of the 38 pan-CoV–positive samples, 18 were selected for partial RNA-dependent RNA polymerase (RdRp), spike (S1) (*14*), and membrane (M) sequencing (Table 1). All samples were negative for PRCV and TGEV by RT-PCR, ruling out co-infection with PEDV and TGEV or PRCV (*15*).

**Table 1:** Distribution of test results of samples from pig farms in study of swine enteric coronaviruses in northern Italy, 2007–2014*

Sample no.	Farm no.	Year	Region	EM	PEDV ELISA	Pan-CoV RT-PCR	TGEV/ PRCV S1	Sequences		
								RdRp	S1	M
222654	1	2007	Emilia Romagna	–	+	+	NA	NA	NA	NA
1448	2	2007	Emilia Romagna	–	+	–	NA	NA	NA	NA
19908	3	2007	Emilia Romagna	–	+	+	–	Cluster I	Cluster I	Cluster I
70323	4	2007	Lombardia	+	+	+	NA	NA	†	NA
114372	5	2007	Lombardia	+	+	+	NA	NA	NA	NA
200079	6	2007	Lombardia	–	+	+	NA	NA	†	NA
320855/5	7	2007	Lombardia	+	+	+	–	Cluster I	Cluster I	Cluster I
320855/6	7	2007	Lombardia	+	+	+	NA	†	†	NA
3936/1	8	2008	Lombardia	–	+	+	–	Cluster I	Cluster I	Cluster I
3936/2	8	2008	Lombardia	–	+	+	NA	†	NA	NA
29177	9	2008	Veneto	+	+	+	–	Cluster I	Cluster I	Cluster I
43853	10	2008	Lombardia	+	+	–	NA	NA	NA	NA
7239‡	11	2009	Lombardia	–	+	+	–	Cluster I	Cluster I	Cluster I
20001	12	2009	Lombardia	–	+	+	–	Cluster I	Cluster I	Cluster I
20416	13	2009	Lombardia	–	+	+	NA	†	†	NA
22603	14	2009	Lombardia	–	+	+	–	Cluster I	Cluster I	Cluster I
26199/2	15	2009	Lombardia	–	+	–	NA	NA	NA	NA
87565	16	2009	Emilia Romagna	–	+	+	NA	NA	†	NA
111357/7	17	2009	Lombardia	NA	+	–	NA	NA	NA	NA
137442	18	2009	Lombardia	+	+	+	–	Cluster II	Cluster II	Cluster II
205396	19	2009	Lombardia	+	+	–	NA	NA	NA	NA
208995	20	2009	Lombardia	+	–	+	NA	†	NA	NA
213306‡	21	2009	Lombardia	+	+	+	–	Cluster II	Cluster II	Cluster II
244945	22	2009	Emilia Romagna	+	+	–	NA	NA	NA	NA
245242	22	2009	Emilia Romagna	+	+	+	NA	†	NA	NA
274771	23	2009	Veneto	+	–	–	NA	NA	NA	NA
307121	24	2009	Emilia Romagna	+	+	+	–	Cluster II	Cluster II	Cluster II
315994	25	2009	Lombardia	+	–	–	NA	NA	NA	NA
320695	26	2009	Lombardia	+	+	+	NA	NA	†	†
320825	26	2009	Lombardia	+	+	+	–	Cluster II	Cluster II	Cluster II
324345	27	2009	Lombardia	+	+	+	NA	†	†	†
324374	27	2009	Lombardia	+	+	+	NA	†	†	†
324397	27	2009	Lombardia	+	+	+	–	Cluster II	Cluster II	Cluster II
324507/1	28	2010	Lombardia	+	+	+	–	Cluster II	Cluster II	Cluster II
324507/2	28	2010	Lombardia	+	+	+	NA	NA	†	†
324507/3	28	2010	Lombardia	+	+	+	NA	NA	NA	†
324507/4	28	2010	Lombardia	+	+	+	NA	NA	†	†
5448/2	29	2011	Emilia Romagna	NA	+	–	NA	NA	NA	NA
28607	30	2012	Lombardia	–	+	+	NA	NA	†	†
29742	30	2012	Lombardia	+	+	+	–	Cluster II	Cluster II	Cluster II
30917	31	2012	Lombardia	+	+	–	NA	NA	NA	NA
35621/1	32	2012	Lombardia	+	+	–	NA	NA	NA	NA
35621/2	32	2012	Lombardia	–	+	+	NA	NA	NA	NA
41906	33	2012	Lombardia	–	+	+	NA	NA	NA	NA
44833	34	2012	Lombardia	NA	+	+	–	Cluster II	Cluster II	Cluster II
67322	8	2012	Lombardia	–	+	+	NA	NA	NA	†
273992	35	2012	Lombardia	–	+	+	–	Cluster II	Cluster II	Cluster II
32961	36	2013	Piemonte	–	+	–	NA	NA	NA	NA
32963	36	2013	Piemonte	+	+	–	NA	NA	NA	NA
178509	37	2014	Emilia Romagna	NA	NA	+	–	Cluster III	Cluster III	Cluster III
200885	38	2014	Emilia Romagna	+	+	+	–	Cluster III	Cluster III	Cluster III

*Cluster I represents strains circulating from 2007 through mid-2009; cluster II represents strains circulating from mid-2009 through 2012; and cluster III represents strains circulating since 2014. EM, electron microscopy; M, membrane; pan-CoV RT-PCR, pan-coronavirus reverse transcription PCR; PEDV, porcine epidemic diarrhea virus; PRCV, porcine respiratory coronavirus; RdRp, RNA-dependent RNA polymerase; S1, spike; TGEV, transmissible gastroenteritis virus; +, positive; –, negative; NA, not tested or sequenced. †Sequence available but not included in this study. ‡Samples selected for whole genome sequencing.

**Figure 1:**
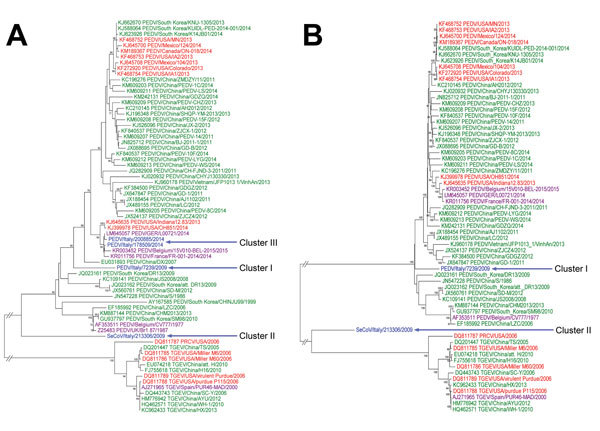
Phylogenetic analyses of swine enteric coronaviruses in Italy. A) Analysis performed on the basis of the nucleotide sequence of the complete spike (S1) gene of 4 representative strains from the 3 clusters and B) whole genome of 2 positive strains from clusters I and II. Cluster I represents strains circulating from 2007 through mid-2009; cluster II represents strains circulating from mid-2009 through 2012; and cluster III represents strains circulating since 2014. Bootstrap values >70% (1,000 replicates) are indicated. Reference sequences are identified by GenBank accession no. and strain name. The strains from this study are represented in blue; strains from China are green; strains from North America are red; and strains from Europe are purple. PEDV, porcine epidemic diarrhea virus; PRCV, porcine respiratory coronavirus; TGEV, transmissible gastroenteritis virus; SeCoV, swine enteric coronavirus.

On the basis of the partial sequences from RdRp and the S1 and M genes, the strains from Italy clustered into 3 temporally divided groups, suggesting 3 independent virus entries. Cluster I represents strains circulating from 2007 through mid-2009; cluster II represents strains circulating from mid-2009 through 2012; and cluster III represents strains circulating since 2014 (Technical Appendix Figure 2, panels A–C). Cluster I was identified in Emilia Romagna (n = 1), Lombardia (n = 5), and Veneto (n = 1). Cluster II was identified in Emilia Romagna (n = 1) and Lombardia (n = 8). Cluster III was identified in Emilia Romagna at 2 swine farms. To help explain the temporal clustering, a single S1 gene segment was sequenced from clusters I and II (PEDV/Italy/7239/2009 and SeCoV/Italy/213306/2009, respectively). Because of the recent outbreak of PEDV in Europe, the 2 positive samples from cluster III (PEDV/Italy/178509/2014 and PEDV/Italy/200885/2014) were sequenced (Figure 1, panel A).

**Figure 2:**
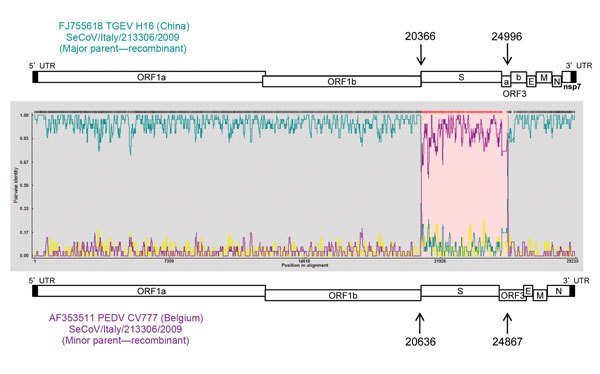
Potential recombination points in the SeCoV strains in study of swine enteric coronaviruses in Italy. The potential parent strains H16 (TGEV) and CV777 (PEDV) are shown in teal and purple, respectively. Arrows indicate recombinant breakpoints. PEDV, porcine epidemic diarrhea virus; TGEV, transmissible gastroenteritis virus; seCoV, swine enteric coronavirus.

One strain from each cluster was selected for whole genome sequencing (Technical Appendix). Unfortunately, the whole genome was obtained from only clusters I and II (PEDV/Italy/7239/2009 and SeCoV/Italy/213306/2009, respectively; Figure 1, panel B). Recombination analysis was conducted on the 2 whole genomes and was not detected in PEDV/Italy/7239/2009. Recombination was detected in SeCoV/Italy/213306/2009 at position 20636 and 24867 of PEDV CV777 and at position 20366 and 24996 of TGEV H16 (Figure 2), suggesting the occurrence of a recombination event between a PEDV and a TGEV. The complete S gene of SeCoV/Italy/213306/2009 shared 92% and 90% nt identity with the prototype European strain PEDV CV777 and the original highly virulent North American strain Colorado 2013, respectively, and the remaining genome shared a 97% nt identity with the virulent strains TGEV H16 and TGEV Miller M6. Whole-genome analysis of PEDV/Italy/7239/2009 showed that it grouped with the global PEDV strains (*6*) and shared ≈97% nt identity with PEDV strains CV777, DR13 virulent, and North American S-INDEL strain OH851 (Table 2).

**Table 2:** Nucleotide identities of strains PEDV/Italy/7239/2009 and SeCoV/Italy/213306/2009, representative of clusters I and II, respectively, in study of swine enteric coronaviruses in Italy*

Strain identification	ORF1			Spike			ORF3			Envelope			Membrane			Nucleocapsid	
	I	II		I	II		I	II		I	II		I	II		I	II
PEDV/Belgium/CV777/1977	97.3	57.8		96.3	92.7		98.1	43.6		97.6	43.0		97.6	55.9		96.6	42.5
PEDV/South Korea/DR13 vir/2009	98.1	58.0		97.3	93.1		99.3	43.7		99.6	43.0		97.7	55.6		97.6	43.3
PEDV/USA/Colorado/2013	98.0	57.9		94.6	90.7		98.6	44.0		99.2	43.0		97.8	55.3		96.8	43.4
PEDV/USA/OH851/2014	98.1	57.9		96.9	91.9		98.7	43.8		99.2	43.0		97.7	55.5		96.7	43.4
PEDV/L00721/GER/2014	98.0	57.9		97.0	92.0		98.6	43.7		99.2	43.0		97.8	55.3		96.8	43.4
TGEV/USA/Miller M6/2006	57.9	96.8		52.0	52.5		40.0	89.1		42.6	96.4		56.1	97.1		43.1	96.3
PRCV/USA/ISU-1/2006	58.0	96.5		47.7	48.2		53.0	76.6		43.8	96.0		56.2	96.3		42.8	95.7

*Cluster I represents strains circulating from 2007 through mid-2009; cluster II represents strains circulating from mid-2009 through 2012. Ger, Germany; ORF, open reading frame; PRCV, porcine respiratory coronavirus; PEDV, porcine epidemic diarrhea virus; SeCoV, swine enteric coronavirus; TGEV, transmissible gastroenteritis virus

## Conclusions

During 2007–2014, most (92%) samples collected from the Po Valley in Italy were positive for PEDV by ELISA; only 72% were positive by pan-CoV PCR. However, because we were investigating the presence of PEDV or TGEV in samples with clinical signs of diarrhea, the high occurrence of PEDV may not reflect the actual prevalence of PEDV in Italy. The increased percentage of PEDV found in samples tested by ELISA, compared with the proportion found by PCR, may be explained by the number of ambiguous bases in the pan-CoV primers; the ambiguous bases severely reduce the efficiency of the reaction. The swine enteric CoV strains from Italy in our study, including the recombinant strain, were reported in pigs with mild clinical signs, indicating that PEDV and SeCoV have been circulating in Italy and likely throughout Europe for multiple years but were underestimated as a mild form of diarrhea. 

To understand the evolution of PEDV in Italy, the partial RdRp, S, and M genes were sequenced from 18 samples and grouped in 3 different temporal clusters. Cluster I (2007–mid 2009) resembles the oldest PEDV strains; cluster II resembles a new TGEV and PEDV recombinant variant; and cluster III, identified from 2 pig farms in northern Italy in 2014, resembles the PEDV S-INDEL strains identified in Germany, France, Belgium, and the United States. The >99.3% nt identity of the S1 gene within cluster III and in previously identified strains could suggest the spread of the S-INDEL strain into Europe. However, directionality of spread cannot be determined because of a lack of global and temporal PEDV sequences.

Although our findings could indicate 3 introductions of PEDV in Italy, the results more likely suggest the high ability of natural recombination among CoVs and the continued emergence of novel CoVs with distinct pathogenic properties. Further investigation is needed to determine the ancestor of the SeCoV strain or to verify whether the recombinant virus was introduced in Italy. Recombinant SeCoV was probably generated in a country in which both PEDV and TGEV are endemic, but because the presence of these viruses in Europe is unclear and SeCoV has not been previously described, it is difficult to determine the parental strains and geographic spread of SeCoV. Future studies are required to describe the pathogenesis of SeCoV and its prevalence in other countries.

**Technical Appendix.** Additional methods, tables, and figures from study of swine enteric coronaviruses in Italy.
