# Supplementary material for: Porcine Epidemic Diarrhea Virus and Discovery of a Recombinant Swine Enteric Coronavirus, Italy
Source: Emerg Infect Dis. 2016 Jan;22(1):83–7. doi: 10.3201/eid2201.150544 (PMC4696687; doi:10.3201/eid2201.150544)
Supplement: Supplementary file 1 — Technical Appendix. Additional methods, tables, and figures from study of swine enteric coronaviruses in Italy. [file 15-0544-Techapp-s1.pdf]

Article DOI: http://dx.doi.org/10.3201/eid2201.150544

Porcine Epidemic Diarrhea Virus and Discovery of a Recombinant Swine Enteric Coronavirus, Italy

Technical Appendix

Additional Methods and Details

Clinical Samples

Fecal and intestinal samples were collected from pigs with clinical signs of diarrhea. The specimens were homogenized in phosphate-buffered saline (10% wt/vol), centrifuged at 2,000 g for 10 min, and the supernatant was poured into a new tube and stored at –80°C.

Virus Detection

Negative staining electron microscopy was performed as previously described (*1*). A double antibody sandwich ELISA was performed by using monoclonal antibodies (*2*). Virus isolation of Porcine epidemic diarrhea virus (PEDV) was attempted on Vero cells in minimum essential medium Eagle (MEM) (Sigma-Aldrich, Saint Louis, Missouri, USA), in the presence of trypsin as previously described (*3*).

RNA Extraction and Reverse Transcription-PCR (RT-PCR)

RNA was extracted from the supernatant by using the TRIzol method (Invitrogen, Carlsbad, CA, USA), and RT-PCR was performed by using the QIAGEN OneStep RT-PCR kit (QIAGEN, Hilden, Germany), performed with the following cycling conditions: 50°C for 30 min and 95°C for 15 min for the RT reaction, followed by 40 cycles of amplification at 95°C for 30 s, 50°C for 30 s, and 72°C for 1 min, with a final extension at 72°C for 10 min. Samples were tested with the pan-CoV primers for the RNA-dependent RNA polymerase (RdRp) gene (*4*). If the samples were positive by the pan-CoV RT-PCR, a larger fragment of the RdRp (349 nt), the

spike (553 nt), and M (439 nt) genes were amplified by RT-PCR (Technical Appendix Table 1). The M gene primers were designed from the conserved regions of 30 Transmissible gastroenteritis virus (TGEV), Porcine respiratory coronavirus (PRCV), and PEDV sequences present in the GenBank database while the RdRp primers were designed from 41 *alpha*, *beta*, *gamma*, and *delta* CoVs sequences from GenBank (Technical Appendix Table 1). Amplification of partial S1 (553 nt) was performed by primers described by Kim et al. 2001 (*5*), and the S1 segment (2785 nt) was designed by using the conserved regions of 18 available PEDV sequences (Technical Appendix Table 1). The amplicons were sequenced with the initial amplification primers by using ABI PRISM 3130 Automated Capillary DNA Sequencer (Applied Biosystems, Waltham, Massachusetts, USA). The 18 sequenced samples were then tested by PCR for TGEV and PRCV by using the previously described RT-PCR kit and thermal cycling conditions (*6*). The phylogenetic trees for the partial RdRp, S1, and M genes, the complete S1 gene and full-length genomes were constructed by using the neighbor-joining method p-distance model and bootstrap test of 1,000 replicates in MEGA 5 (http://www.megasoftware.net/).

Next Generation Sequencing Assembly

Whole genome sequencing was performed on 1 strain from each genetic cluster because of limited available volume of sample. Seven milliliters of a 20% fecal homogenate were ultracentrifuged on 5 ml of 30% sucrose cushion at 35,000 rpm for 1 h at 4°C. The pellet was resuspended in 1x TE (10 mM Tris-HCl, 1 mM EDTA, pH 8) and treated with DNase (RNase-free DNase set, QIAGEN, Hilden, Germany). Purified viral RNA was extracted by using the protocol described above. Viral cDNA synthesis and whole genome amplification of nucleic acids were performed as described by Victoria and colleagues (*7*). The sequencing libraries were prepared by using the NEXTERA-XT DNA library Preparation kit (Illumina Inc. San Diego, CA, USA), and purified according to the manufacturer protocol and sequenced on an Illumina MiSeq by using the Miseq Reagent Kit v2, 250-cycle paired-end run (Illumina Inc. San Diego, CA, USA). Reads were assembled by using PEDV or TGEV reference genomes. When mean coverage was <5 or polymorphisms were present in more than 20% of the reads, Sanger sequencing was performed by primers (Technical Appendix Table 2).

Nucleotide Sequence Accession Numbers

Partial and complete genome sequences obtained in this study were deposited in GenBank under accession nos. KT027383-KT027398 (RdRp), KT027399-KT027414 (S1), KT027415-KT027430 (M), KR061458 (PEDV/Italy/7239/2009) and KR061459 (SeCoV/Italy/213306/2009).

Recombination Analysis

Recombination breakpoints were determined by using the Recombination Detection Program (RDP) 4.43 (http://web.cbio.uct.ac.za/~darren/rdp.html), with BOOTSCAN, GENECONV, MAXCHI, CHIMAERA, SISCAN, 3SEQ and LARD. Statistical significance was determined by using *p*-values <0.05.

References

1. Martelli P, Lavazza A, Nigrelli AD, Merialdi G, Alborali LG, Pensaert MB. Epidemic of diarrhoea caused by porcine epidemic diarrhoea virus in Italy. Vet Rec. 2008;162:307–10. PubMed http://dx.doi.org/10.1136/vr.162.10.307

2. Sozzi E, Luppi A, Lelli D, Moreno Martin A, Canelli E, Brocchi E, et al. Comparison of enzyme-linked immunosorbent assay and RT-PCR or the detection of porcine epidemic diarrhoea virus. Res Vet Sci. 2010;88:166–8. PubMed http://dx.doi.org/10.1016/j.rvsc.2009.05.009

3. Hofmann M, Wyler R. Propagation of the virus of porcine epidemic diarrhea in cell culture. J Clin Microbiol. 1988;26:2335–9. PubMed

4. Lelli D, Papetti A, Sabelli C, Rosti E, Moreno A, Boniotti MB. Detection of coronaviruses in various bat species in Italy. Viruses. 2013;5:2679–89. PubMed http://dx.doi.org/10.3390/v5112679

5. Kim SY, Song DS, Park BK. Differential detection of transmissible gastroenteritis virus and porcine epidemic diarrhea virus by duplex RT-PCR. J Vet Diagn Invest. 2001;13:516–20. PubMed http://dx.doi.org/10.1177/104063870101300611

6. Kim L, Chang KO, Sestak K, Parwani A, Saif LJ. Development of a reverse transcription–nested polymerase chain reaction assay for differential diagnosis of transmissible gastroenteritis virus and porcine respiratory coronavirus from feces and nasal swabs of infected pigs. J Vet Diagn Invest. 2000;12:385–8. PubMed http://dx.doi.org/10.1177/104063870001200418

7. Victoria JG, Kapoor A, Dupuis K, Schnurr DP, Delwart EL. Rapid identification of known and new RNA virus from animal tissues. PLoS Pathog. 2008;4:e1000163. PubMed http://dx.doi.org/10.1371/journal.ppat.1000163

Technical Appendix Table 1. Primers used for sequencing for the S1, M and RdRp genes*

Primers	Oligonucleotide sequence (5′-3′)	Position†
PEDV_S_1F	GGTAAGTTGCTAGTGCGTAA	20570–50589 nt
PEDV_S_1R	TCCCATGTTATGCCGACAA	21116–21134 nt
PEDV_S_2F	TAATGATGTTACAACAGGTC	21039–21058 nt
PEDV_S_2R	AGCAGTATGAAGTACAATTG	21629–21648 nt
PEDV_S_3F	GGCTCTGAGGTTTAATATTA	21579–21598 nt
PEDV_S_3R	GGTAAATTGTCTAGTGTCAA	22259–22278 nt
PEDV_S_4F	TGAGTTGATTACTGGCAC	22503–22520 nt
PEDV_S_4R	GCCATTAGTAACCACTTTAT	23336–23355 nt
M_1F	AYCTTRSAAACTGGAAYTTC	25722–25741 nt
M_1R	ACATAGWAAGCCCAWCCAGT	26258–26277 nt
RdRp gene CoV_8F	GGNTGGGAYTAYCCNAARTGYGA	14426–14448 nt
RdRp gene CoV_10R	TGYTGNGARCARAAYTCRTG	15008–15027 nt

*M, membrane; RdRp, RNA-dependent RNA polymerase; S1, spike.
†Nucleotide position refers to PEDV CV777(AF353511).

Technical Appendix Table 2. Primers used to close the genome

Primers	Oligonucleotide sequence (5′-3′)	Position*
213306_1_for	AGTAAAGTGAGTGTAGCGT	1 –19 nt
213306_1_rev	ACAAGGGCATCTAAGAGTA	1014–1032 nt
213306_2_for	TATGGGAAATGGTGACTGT	957–975 nt
213306_2_rev	GATGTCGTAAGATGGAACT	2055–2073 nt
213306_3_for	GATTGGTAATGGTGTGAAAGT	2517–2537 nt
213306_3_rev	CATTACCAGGAGCAATAGATT	4466–4486 nt
213306_4_for	TCATTGTCACACATACTACAG	5279–5299 nt
213306_4_rev	ACTACAATCACAAACTCAGC	6391–6410 nt
213306_5_for	ATAGTAGTCAAGAGGTTCTC	6809–6828 nt
213306_5_rev	CATAGCAGCAGATTCAAAGT	8675–8692 nt
213306_6_for	TACATGGGCTAAAACTAACAGT	9624–9645 nt
213306_6_rev	GAGGGCTAACATCATTTTTCTTA	11046–11068 nt
213306_7_for	TAGTGGAAAGGCTCTTATG	11634–11652 nt
213306_7_rev	TGACAATGTAGTAGGCATC	12480–12498 nt
213306_8_for	CGTTGCGTGTATTGGTAAA	12416–12434 nt
213306_8_rev	CGTCATTGGTATTGTCATAG	13237–13256 nt
213306_9_for	AGACAGTAAAACCAGGTCA	13507–13525 nt
213306_9_rev	ATGGTGTGATAGACATAATGAA	15201–15222 nt
213306_10_for	AGGCTATCACACTCTATTTTG	17078–07098 nt
213306_10_rev	CACAACTTTGGACACAACAT	19348–19367 nt
213306_11_for	CTCTATCACATAACTCAGTC	20167–20186 nt
213306_11_rev	TAGGTAGATAACCACCCAA	20499–20517 nt
213306_12_for	GTGACCACACCTATGAC	25375–25391 nt
213306_12_rev	CCAAGCGTAGTAGGTTTG	26812–26829 nt
213306_13_for	GTAGAGGTGATGTGACAA	27171–27188 nt
213306_13_rev	ATTGTTGGCTCGTCATAG	28017–28034 nt
7239_1_for	ATCTAYGGATAGTTAGCTCT	1–20 nt
7239_1_rev	TCACCAACAGTCCAATGA	1027–1044 nt
7239_2_for	GTGACGGGTTTTACAGAC	1977–1994 nt
7239_2_rev	ACAAAAGAAGTTTCAATGACGC	2790–2811 nt
7239_3_for	TGTCCAGGAAGATGTTCA	4672–4689 nt
7239_3_rev	TCCAATTTGTTGTCCATAAGTA	5233–5254 nt
7239_4_for	GTAATGGTAACGGTGTTGT	5913–5931 nt
7239_4_rev	GGAAAGGCATCACATTAC	6591–6608 nt
7239_5_for	GAAGTTGGTAATGTTGTCAAAC	7031–7052 nt
7239_5_rev	TATCACTGCTAACCTGAGTA	7921–7940 nt
7239_6_for	CTATGACCTACTGTCGT	8442–8458 nt
7239_6_rev	TAGGCGTATGGACATTGT	9543–9560 nt
7239_7_for	AGATTTCTTCCGTTCAGTCTA	10995–11015 nt
7239_7_rev	CACAAGCGCCTACCTTA	12996–13012 nt
7239_8_for	CTAAGCGTAACATCCTGC	14150–14167 nt
7239_8_rev	TGGAGTGATGGACAAAATGAAT	15474–15495 nt
7239_9_for	CAAGGAGGAGAGCGTTA	15774–15790 nt
7239_9_rev	GAAACCCATAAACGAGATAAC	17305–17325 nt

Primers	Oligonucleotide sequence (5′-3′)	Position*
7239_10_for	AGGGATCACTTAGCCTTAA	17891–17909 nt
7239_10_rev	CAAGGTTCTAAACACATACG	19765–19784 nt
7239_11_for	CTAATGTGCTGGGTGTTTC	23230–23248 nt
7239_11_rev	ATAAACTCTATACAAACGCCCTA	25579–25601 nt
7239_12_for	CAGTGTAGTTGAGATTGTTGAAC	26738–26760 nt
7239_12_rev	TAGGCTCGTCAAGCGGAT	27918–27935 nt

*Nucleotide position refers to complete genome of the samples 7239 and 213306.

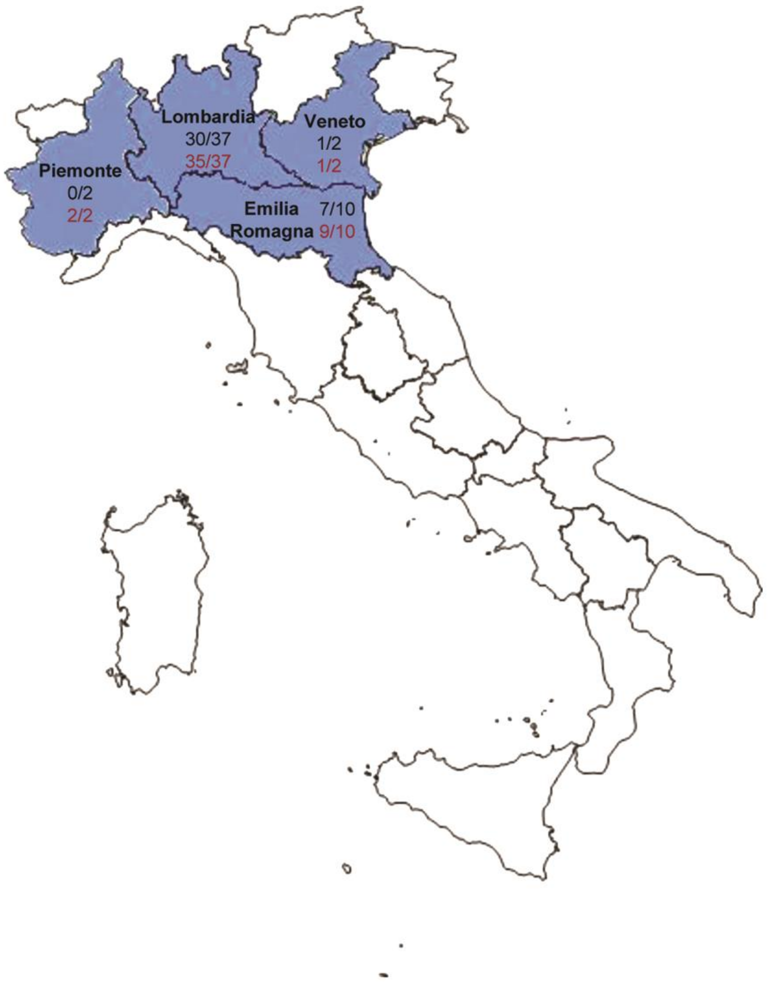

Technical Appendix Figure 1. Geographic distribution of the ELISA and pan-CoV RT-PCR results (red and black, respectively) from this study.

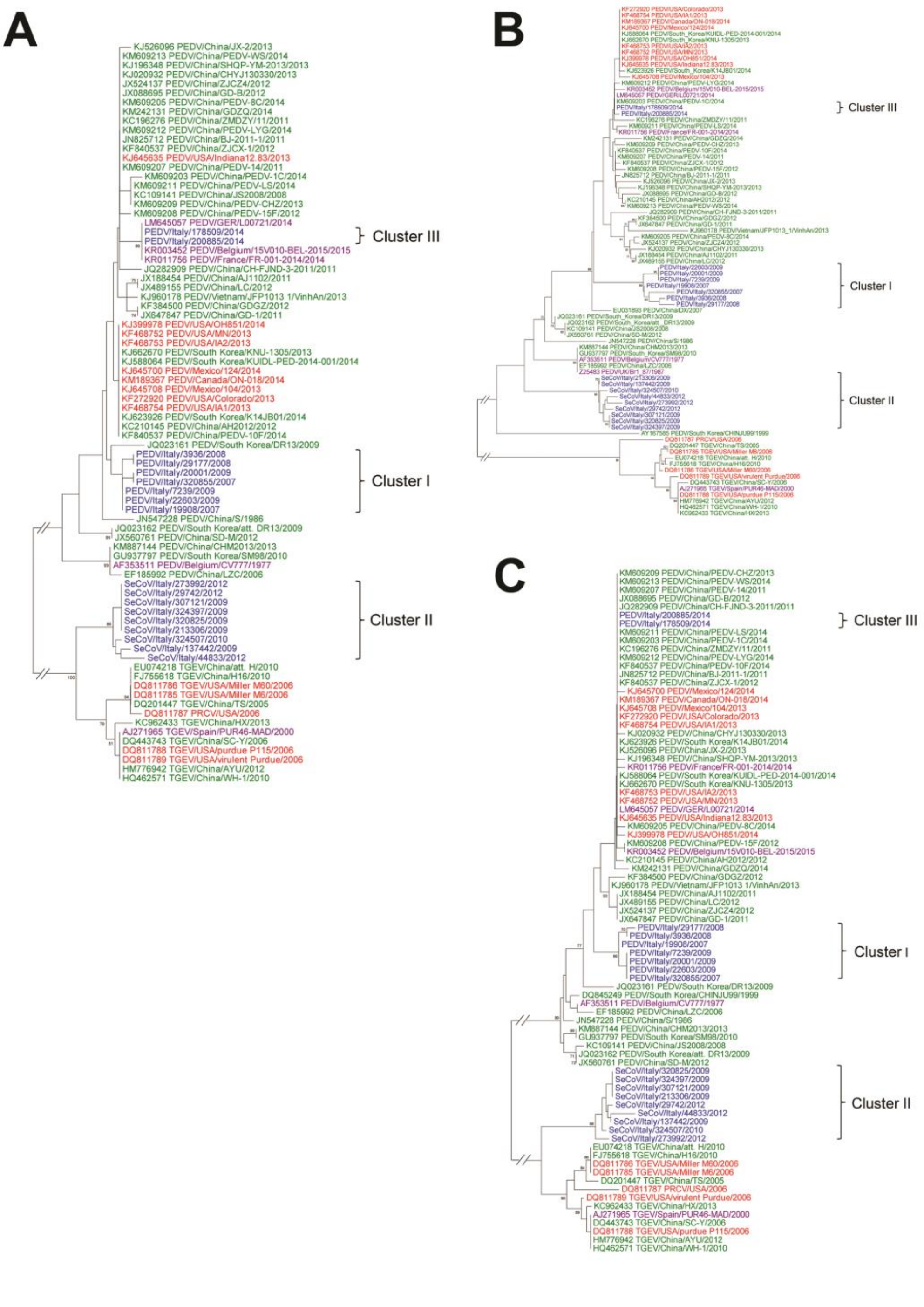

Technical Appendix Figure 2. Phylogenetic trees from the study of porcine epidemic diarrhea virus and a recombinant swine enteric coronavirus. A) Partial RNA-dependent RNA polymerase gene; B) spike (S1) gene; and C) membrane (M) gene. Bootstrap values >70% (1,000 replicates) are indicated. Reference sequences are identified by GenBank accession no. and strain name. The 18 strains from Italy identified from this study are represented in blue; strains from China are green; strains from America are red; and

strains from Europe are purple. PEDV, porcine epidemic diarrhea virus; PRCV, porcine respiratory coronavirus; TGEV, transmissible gastroenteritis virus; SeCoV, swine enteric coronavirus.
